# Boltzmann sampling from the Ising model using quantum heating of coupled nonlinear oscillators

**DOI:** 10.1038/s41598-018-25492-8

**Published:** 2018-05-08

**Authors:** Hayato Goto, Zhirong Lin, Yasunobu Nakamura

**Affiliations:** 1 Frontier Research Laboratory, Corporate Research & Development Center, Toshiba Corporation, 1, Komukai-Toshiba-cho, Saiwai-ku, Kawasaki, 212-8582 Japan; 2 RIKEN Center for Emergent Matter Science (CEMS), Wako, Saitama, 351-0198 Japan; 3 Research Center for Advanced Science and Technology (RCAST), The University of Tokyo, Meguro-ku, Tokyo, 153-8904 Japan

## Abstract

A network of Kerr-nonlinear parametric oscillators without dissipation has recently been proposed for solving combinatorial optimization problems via quantum adiabatic evolution through its bifurcation point. Here we investigate the behavior of the *quantum bifurcation machine* (*QbM*) in the presence of dissipation. Our numerical study suggests that the output probability distribution of the dissipative QbM is Boltzmann-like, where the energy in the Boltzmann distribution corresponds to the cost function of the optimization problem. We explain the Boltzmann distribution by generalizing the concept of quantum heating in a single nonlinear oscillator to the case of multiple coupled nonlinear oscillators. The present result also suggests that such driven dissipative nonlinear oscillator networks can be applied to Boltzmann sampling, which is used, e.g., for Boltzmann machine learning in the field of artificial intelligence.

## Introduction

Recently, hardware devices designed for combinatorial optimization have attracted much attention. The most well-known example is the quantum annealer developed by D-Wave Systems^1^. The machines are based on quantum annealing or adiabatic quantum computation^2–5^ and are physically implemented with superconducting quantum bits (qubits). Classical annealers with semiconductor classical bits in CMOS devices have also been studied^6^. Both are designed to find the ground state of the Ising model. Such Ising machines are useful in the sense that many combinatorial optimization problems can be transformed into the Ising problem^7^.

Another approach to the physical implementation of Ising machines is based on parametric oscillations, where two stable oscillating states of each parametric oscillator correspond to up and down spins^8–15^. There are two major types of such Ising machines. The first type originally proposed in ref.^8^ uses a network of optical parametric oscillators (OPOs). The threshold of an OPO is determined by one-photon loss and its oscillating states are stabilized by two-photon loss. The coupling between two OPOs is implemented by mutual injection^8,9^ or measurement feedback^10,11^, where the energy of the network is not conserved. Thus, dissipation is indispensable for the first type of Ising machines. The second type originally proposed in ref.^13^ uses a network of nondissipative Kerr-nonlinear parametric oscillators (KPOs). The threshold of a KPO is determined by one-photon detuning and its oscillating states are stabilized by the Kerr effect (resonance frequency shift depending on the oscillation power). The coupling between two KPOs is implemented by photon exchange, where the energy of the network is conserved. Thus, the second type of Ising machines can in principle be operated without dissipation, unlike the first type, and is based on quantum adiabatic evolution. Such machines can be implemented with superconducting circuits, as suggested in ref.^13^, and explicit circuit designs for all-to-all connectivity have recently been proposed in refs^14,15^.

In the present work, we numerically investigate the effects of dissipation on the second type of Ising machines with KPOs. Hereafter, we call this a *quantum bifurcation machine*, or *QbM* for short, because the operation principle is based on a quantum-mechanical bifurcation of the KPO network and is called bifurcation-based adiabatic quantum computation^13^. (We do not use “QBM” because it is often used for quantum Boltzmann machine.) Our simulation results indicate that the probability distributions of the spin configurations in dissipative QbMs are Boltzmann-like with respect to the Ising energies.

To explain the Boltzmann distributions of the spin configurations in dissipative QbMs, here we generalize the concept of *quantum heating*^16–18^ in a single driven dissipative nonlinear oscillator to the case of multiple coupled nonlinear oscillators. The quantum heating is the heating process induced by dissipation in quasienergy levels of driven dissipative quantum nonlinear systems, where the quasienergies are defined as eigenvalues of the system Hamiltonian in a rotating frame. Such phenomena have been studied with a parametrically driven Duffing oscillator^16,17,19^ (essentially the same as a KPO), a driven Jaynes-Cummings model in cavity quantum electrodynamics^20,21^, and an optomechanical system where a driven linear cavity is coupled to a mechanical linear resonator in a nonlinear manner^22^. (The terminology “quantum heating” was introduced in ref.^17^ and therefore is not used in the previous literature^19–21^.) In the previous studies, the Boltzmann distribution of quasienergies close to one of local minima of the Hamiltonian is derived analytically by deriving a local Hamiltonian by linearization around the chosen local minimum. (The quasienergy distribution deviates from the Boltzmann distribution near the top of the potential barrier^19^). In contrast, we use numerically evaluated eigenvalues and eigenstates of the global (exact) Hamiltonian, which is another difference from the previous studies. Our numerical results suggest that the quasienergy distributions in the steady states are also Boltzmann-like. Using this result, we will explain that the generalized quantum heating results in the Boltzmann distributions of the spin configurations in dissipative QbMs.

Although quantum heating causes errors in solving optimization problems, the Boltzmann distribution of the spin configurations means that the QbMs are robust against dissipation, because good approximate solutions are obtained with high probability even in the presence of dissipation. (The robustness has been discussed from different points of view in refs^14^ and^15^.) The present result also suggests that such driven dissipative nonlinear oscillator networks can be applied to Boltzmann sampling from the Ising model. Recently, similar physical implementations of a Boltzmann sampler with Ising machines have also attracted much attention^23–30^ because it is useful for various purposes, such as Boltzmann machine learning in the field of artificial intelligence^31^.

## Results

### Quantum bifurcation machine for the Ising problem with local fields

The QbM proposed in ref.^13^ only applies to the Ising problem *without* local fields. In this paper, we extend the QbM to the Ising problem *with* local fields, which is to find the spin configuration that minimizes the following dimensionless Ising energy:

1 $${E}_{{\rm{Ising}}}({\boldsymbol{s}})=-\,\frac{1}{2}\sum _{i=1}^{N}\sum _{j=1}^{N}{J}_{i,j}{s}_{i}{s}_{j}+\sum _{i=1}^{N}{h}_{i}{s}_{i}$$

where *s*_*i*_ is the *i*-th Ising spin, which takes +1 (up) or −1 (down), *N* is the total number of Ising spins, ${\boldsymbol{s}}=({s}_{1}\,{s}_{2}\cdots {s}_{N})$ is the vector representation of a spin configuration, and {*J*_*i,j*_} and {*h*_*i*_} are the dimensionless parameters corresponding to the coupling coefficients and local fields, respectively. Note that {*J*_*i,j*_} satisfies *J*_*i,j*_ = *J*_*j,i*_ and *J*_*i,i*_ = 0. This extension is significant because many applications such as the traveling salesman problem and Boltzmann machine learning require local fields^7,23,30,31^.

For a given instance of the Ising problem, the extended QbM is defined by the following Hamiltonian in a frame rotating at half the pump frequency, *ω*_*p*_/2, of the parametric drive and in the rotating-wave approximation^13^:

2 $$H=\hslash \sum _{i=1}^{N}{H}_{{\rm{K}}{\rm{P}}{\rm{O}}}^{(i)}-\hslash {\xi }_{0}\sum _{i=1}^{N}\sum _{j=1}^{N}{J}_{i,j}{a}_{i}^{\dagger }{a}_{j}+\hslash {\xi }_{0}A(t)\sum _{i=1}^{N}{h}_{i}({a}_{i}^{\dagger }+{a}_{i})$$

3 $${H}_{{\rm{K}}{\rm{P}}{\rm{O}}}^{(i)}=\hslash [\frac{K}{2}{a}_{i}^{\dagger 2}{a}_{i}^{2}+{\rm{\Delta }}{a}_{i}^{\dagger }{a}_{i}-\frac{p(t)}{2}({a}_{i}^{\dagger 2}+{a}_{i}^{2})]$$

where ${a}_{i}^{\dagger }$ and *a*_*i*_ are the creation and annihilation operators for the *i*-th KPO, *K* is the Kerr coefficient, Δ is the detuning frequency defined by ${\rm{\Delta }}={\omega }_{{\rm{KPO}}}-{\omega }_{p}/2$ (*ω*_KPO_ is the resonance frequency of the KPOs at low powers), *p*(*t*) is the time-dependent pump amplitude, and *ξ*_0_ is a constant parameter with the dimension of frequency. Here, we assume for simplicity that *K*, Δ, and *ξ*_0_ are positive. If *K* is negative, as in the case of superconducting Josephson parametric oscillators^32^, we set *p*(*t*), Δ, and *ξ*_0_ to negative values by flipping the signs. Then, we obtain the same result. The physical meaning of the third term in Eq. (2), which is added for the extension, is the external drive of KPOs at *ω*_*p*_/2, where *ξ*_0_*A*(*t*)*h*_*i*_ is the time-dependent amplitude of the external drive for the *i*-th KPO and *A*(*t*) is a dimensionless positive parameter defined such that *A* ≈ 0 when *p* << Δ and $A\approx {\alpha }_{0}=\sqrt{(p-{\rm{\Delta }})/K}$ when *p* >> Δ. Here, *α*_0_ is the magnitude of the amplitudes of the two stable oscillating states of each KPO^13^. This is derived by minimizing $\langle \alpha |{H}_{{\rm{KPO}}}^{(i)}|\alpha \rangle =\hslash [K{|\alpha |}^{4}/2+({\rm{\Delta }}-p){|\alpha |}^{2}]$ with respect to the amplitude, *α*, of the coherent state |*α*〉. (A coherent state |*α*〉 is defined as the eigenstate of an annihilation operator: ${a}_{i}|\alpha \rangle =\alpha |\alpha \rangle$^33^).

To find the ground state of the Ising model via quantum adiabatic evolution, we initialize all the KPOs in the “vacuum” state and gradually increase the pump amplitude *p*(*t*) from zero to a sufficiently large value compared to Δ and *ξ*_0_. To satisfy the initial condition that the vacuum state is the ground state of the initial Hamiltonian, Δ is set such that the matrix *M* defined by *M*_*i,i*_ = Δ and *M*_*i,j*_ = − *ξ*_0_*J*_*i,j*_ (*i* ≠ *j*) is positive semidefinite^13^. If the variation of *p*(*t*) is sufficiently slow, the final state will become the ground state of the final Hamiltonian by the quantum adiabatic theorem. In the following, we explain that this final state provides the solution of the given problem, namely, the ground state of the given Ising model.

When *p*(*t*) becomes sufficiently large and *ξ*_0_ is sufficiently small, low energy states for the final Hamiltonian can be approximately expressed with the vectors $|{\boldsymbol{s}}\rangle :=|{s}_{1}{\alpha }_{0}\rangle |{s}_{2}{\alpha }_{0}\rangle \cdots |{s}_{N}{\alpha }_{0}\rangle$, where ${s}_{i}=\pm \,1$ is the sign of the oscillation amplitude of the *i*-th KPO. (As mentioned above, $|\pm {\alpha }_{0}\rangle$ minimize the dominant term of the Hamiltonian, namely, the first term in Eq. (2).) Using the vectors |***s***〉, the Hamiltonian for low energy states is expressed as a diagonal matrix (off-diagonal elements are dropped assuming that $\langle -{\alpha }_{0}|{\alpha }_{0}\rangle ={e}^{-2{\alpha }_{0}^{2}} <  < 1$) with the following diagonal elements (eigenenergies):

4 $$\langle {\bf{s}}|H|{\bf{s}}\rangle =\hslash \sum _{i=1}^{N}(\frac{K}{2}{\alpha }_{0}^{4}+{\rm{\Delta }}{\alpha }_{0}^{2}-p{\alpha }_{0}^{2})+2\hslash {\xi }_{0}{\alpha }_{0}^{2}(-\frac{1}{2}\sum _{i=1}^{N}\sum _{j=1}^{N}{J}_{i,j}{s}_{i}{s}_{j}+\sum _{i=1}^{N}{h}_{i}{s}_{i})$$

where we have used *A* ≈ *α*_0_ (this holds for *p* >> Δ). Note that the first term is independent of {*s*_*i*_} and the second term is proportional to the Ising energy *E*_Ising_(***s***) in Eq. (1). This directly shows that the ground state of the final Hamiltonian corresponds to the ground state of the given Ising model. Thus, we obtain the solution of the given problem by measuring the quadrature amplitude defined by ${x}_{i}=({a}_{i}+{a}_{i}^{\dagger })/2$ of the final state and identifying the sign of *x*_*i*_ with the Ising spin *s*_*i*_^13^.

**Figure 1:**
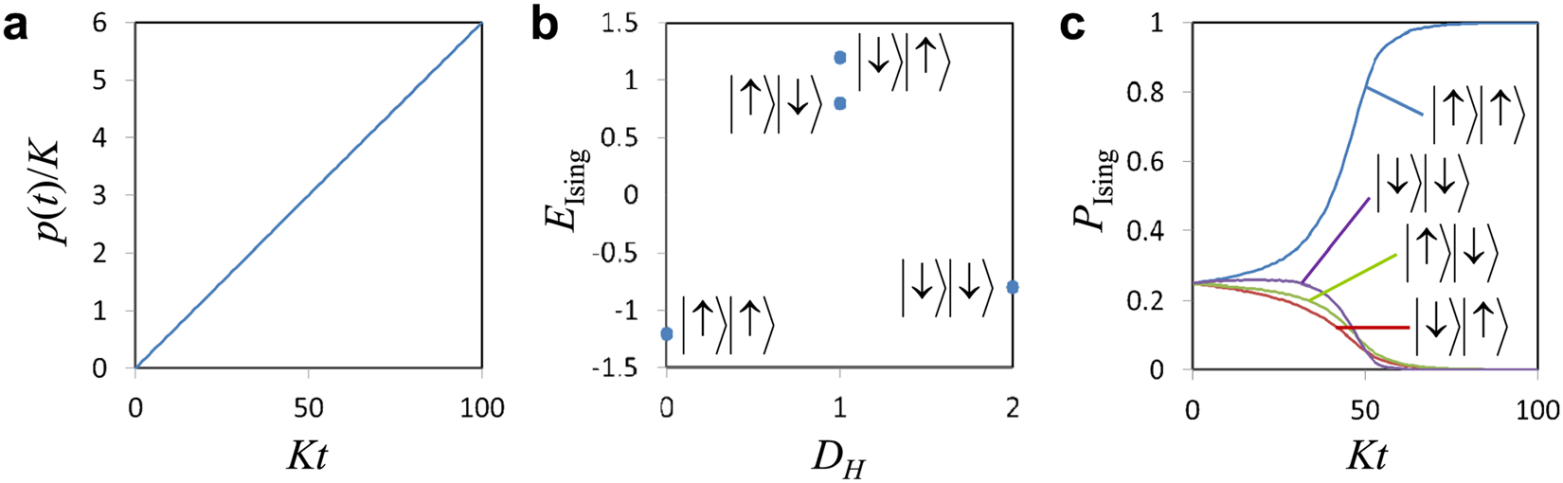
Nondissipative QbM. (**a**) Time-dependent pump amplitude. (**b**) Energy landscape of an instance of the two-spin Ising model. The parameters are ${J}_{1,2}={J}_{2,1}=1$, ${h}_{1}=-\,0.2$, and ${h}_{2}=0$. The horizontal axis represents the Hamming distance ${D}_{H}$ defined as the number of spin flips with respect to the ground state $|\uparrow \rangle |\uparrow \rangle$. (**c**) Time evolutions of the spin configuration probabilities ${P}_{{\rm{Ising}}}({\bf{s}})$ given by Eq. (6).

To verify the validity of the above discussion, we numerically investigate an instance of two KPOs (*N* = 2), where the Schrödinger equation with the Hamiltonian in Eq. (2) is solved numerically. The time-dependent pump amplitude *p*(*t*) is increased linearly, as shown in Fig. 1a. The parameters of the instance are ${J}_{1,2}={J}_{2,1}=1$, *h*_1_ = −0.2, and *h*_2_ = 0, which are set such that two local minima exist in the energy landscape, as shown in Fig. 1b.

In the present numerical study, the Hilbert space is truncated at a “photon” number of 14 for each KPO and *A*(*t*) is set to the following form that satisfies the above conditions (*A* ≈ 0 when *p* << Δ and *A* ≈ *α*_0_ when *p* >> Δ):

5 $$A(t)=\sqrt{\frac{p(t)-{\rm{\Delta }}\,\tanh [p(t)/{\rm{\Delta }}]}{K}}$$

The other parameters are set to Δ = 2*K* and *ξ*_0_ = 0.5 *K*.

Figure 1c shows the time evolutions of the spin configuration probabilities ${P}_{{\rm{Ising}}}({\boldsymbol{s}})$ given by

6 $${P}_{{\rm{I}}{\rm{s}}{\rm{i}}{\rm{n}}{\rm{g}}}({\boldsymbol{s}})={\rm{T}}{\rm{r}}[\rho \prod _{i=1}^{N}{M}^{(i)}({s}_{i})]$$

where *ρ* is the density operator describing the state of the system and

7 $${M}^{(i)}(1)={\int }_{0}^{\infty }d{x}_{i}|{x}_{i}\rangle \langle {x}_{i}|,{M}^{(i)}(\,-\,1)={\int }_{-\infty }^{0}d{x}_{i}|{x}_{i}\rangle \langle {x}_{i}|$$

compose the positive-operator-valued measure for measuring the sign of the quadrature amplitude, *x*_*i*_, of the *i*-th KPO (|*x*_*i*_〉 is the eigenstate of *x*_*i*_). The probabilities *P*_Ising_(***s***) are calculated by the method presented in ref.^13^. As shown in Fig. 1c, the state of the two KPOs finally converges to $|\uparrow \rangle |\uparrow \rangle$, which is the ground state of the given Ising model, as expected.

### Dissipative quantum bifurcation machine

In the presence of dissipation, the time evolution of a QbM is modeled by the following quantum master equation^16–18,34^:

8 $$\dot{\rho }=-\,\frac{i}{\hslash }[H,\rho ]+\kappa (\bar{n}+1)\sum _{i=1}^{N}(2{a}_{i}\rho {a}_{i}^{\dagger }-{a}_{i}^{\dagger }{a}_{i}\rho -\rho {a}_{i}^{\dagger }{a}_{i})+\kappa \bar{n}\sum _{i=1}^{N}(2{a}_{i}^{\dagger }\rho {a}_{i}-{a}_{i}{a}_{i}^{\dagger }\rho -\rho {a}_{i}{a}_{i}^{\dagger })$$

where the dot denotes the time derivative, *κ* is the decay rate of the KPOs characterizing the dissipation, and $\bar{n}={\{\exp [\hslash ({\omega }_{{\rm{p}}}/2)/({k}_{B}T)]-1\}}^{-1}$ is the Planck number at frequency *ω*_p_/2 and temperature *T* (*k*_*B*_ is the Boltzmann constant). While the first term in Eq. (8) describes the unitary time evolution of the system, the other terms are for the non-unitary evolution. In the following, $\bar{n}$ is set to zero assuming a sufficiently low temperature.

We numerically solve the master equation for the same instance as above. In the simulations of dissipative QbMs, we use the following form of *p*(*t*):

9 $$p(t)={p}_{f}\,\tanh (3t/\tau )$$

where *p*_*f*_ is the final value of *p*(*t*) and *τ* is the time at which *p*(*t*) closely approaches *p*_*f*_. The form of *p*(*t*) is chosen such that *p*(*t*) increases linearly with respect to *t* at the initial time and converges to its final value *p*_*f*_. The time *τ* is set to 100 *K*^−1^ in the present work.

**Figure 2:**
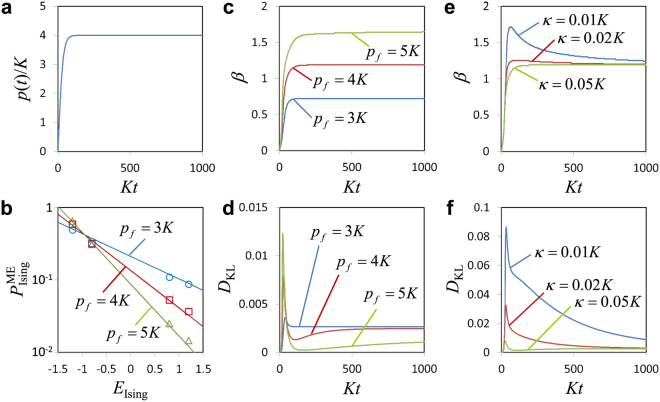
Dissipative QbM. The parameters of the two-spin Ising model are the same as that in Fig. 1. (**a**) Time-dependent pump amplitude $p(t)$ [Eq. (9) with ${p}_{f}=4K$]. (**b**) Probability distributions of the spin configurations. Symbols show the spin configuration probabilities, ${P}_{{\rm{Ising}}}^{{\rm{ME}}}({\bf{s}})$, at the final time ($t=1000{K}^{-1}$), which are obtained with the numerical solution of the quantum master equation [Eq. (8)]. Circles, squares, and triangles correspond to ${p}_{f}=3K$, 4 *K*, and 5 *K*, respectively. The decay rate is set to $\kappa =0.05K$. The lines show the Boltzmann distribution fitting to the simulation results. (**c**) Inverse effective temperature *β* for the three values of ${p}_{f}$ determined by fitting to the instantaneous probability distribution. (**d**) Kullbak-Leibler (KL) divergence ${D}_{{\rm{KL}}}$ minimized for the fitting in (**c**). (**e** and **f**) Time evolutions of *β* and ${D}_{{\rm{KL}}}$ for various values of $\kappa$ (${p}_{f}=4K$).

The simulation results are summarized in Fig. 2. Figure 2a shows the time-dependent pump amplitude *p*(*t*) with *p*_*f*_ = 4 *K*. The symbols in Fig. 2b shows the distribution of the spin configuration probabilities ${P}_{{\rm{Ising}}}^{{\rm{ME}}}({\boldsymbol{s}})$ with respect to the Ising energy ${E}_{{\rm{Ising}}}({\boldsymbol{s}})$ at the final time ($t=1000{K}^{-1}$), where the decay rate is set to $\kappa =0.05K$. The line in Fig. 2b is obtained by fitting the Boltzmann distribution to the simulation results, where the Kullback-Leibler (KL) divergence *D*_KL_ between the two distributions is minimized (see Methods for details). The Boltvzmann distribution is defined by ${P}_{B}({\boldsymbol{s}},\beta )=\exp [-\beta {E}_{{\rm{Ising}}}({\boldsymbol{s}})]/Z(\beta )$, where *β* is the inverse effective temperature and $Z(\beta )=\sum _{{\boldsymbol{s}}}\,\exp [-\beta {E}_{{\rm{Ising}}}({\boldsymbol{s}})]$ is the partition function. In the fitting, *β* is a single fitting parameter. The good fits shown in Fig. 2b indicate that the probability distributions of the spin configurations in the dissipative QbM are Boltzmann-like.

Figures 2c and 2d show the time evolutions of the inverse effective temperature *β* and the minimized KL divergence *D*_*KL*_, respectively. Figure 2c shows that as *p*_*f*_ increases, *β* increases, that is, the effective temperature decreases. Thus, the effective temperature can be controlled by the pump amplitude of the parametric drive. From Figs. 2c and 2d, it is also found that the convergence is slower for larger *p*_*f*_. This is because the potential barriers between different spin configurations are higher for larger *p*_*f*_ (see the next subsection for the potential).

Figures 2e and 2f show similar results for different values of *κ* (*p*_*f*_ = 4 *K*). Figure 2e shows that *β* converges to a single value independent of *κ*, and Fig. 2f supports that the probability distributions approach the Boltzmann distribution. From Figs. 2e and 2f, it is also found that the convergence is faster for larger *κ*, as expected.

**Figure 3:**
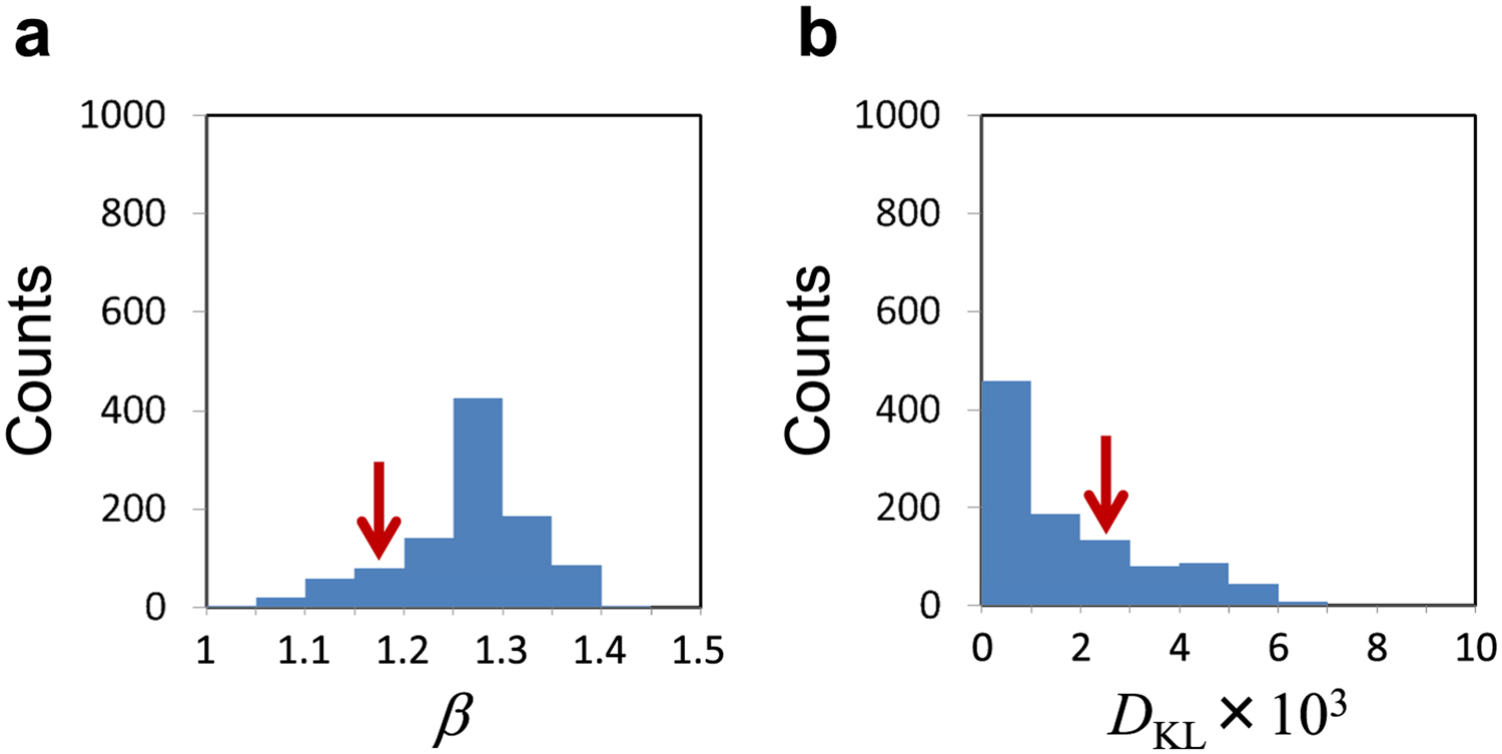
Simulation results of 1000 instances of the two-spin Ising problem. $\{{J}_{i,j}\}$ and $\{{h}_{i}\}$ are chosen randomly from the interval (−1, 1). The time-dependent pump amplitude *p*(*t*) is set as in Fig. 2a. The other parameters are set to ${p}_{f}=4K$ and $\kappa =0.05K$. (**a**) Histogram of the inverse effective temperature *β* determined by fitting to the final probability distribution. (**b**) Histogram of the corresponding Kullbak-Leibler (KL) divergence ${D}_{{\rm{KL}}}$ minimized for the fitting in (**a**). The arrows indicate the results in Fig. 2.

To check that the probability distributions of the spin configurations are also Boltzmann-like for other instances, we perform similar numerical simulations for 1000 instances of the two-spin Ising problem, where their parameters, {*J*_*i,j*_} and $\{{h}_{i}\}$, are chosen randomly from the interval (−1, 1). The other parameters are set to ${p}_{f}=4K$ and $\kappa =0.05K$. The results for *β* and *D*_KL_ are shown in Fig. 3, where the arrows indicate the results of the instance in Fig. 2. The averages and standard deviations are $\beta =1.27\pm 0.07$ and ${D}_{{\rm{KL}}}=(1.7\pm 1.7)\times {10}^{-3}$. The largest value of *D*_KL_ is 6.5 × 10^−3^. On the other hand, when the spin configuration probabilities for each instance are set randomly by choosing four random numbers from the interval (0, 1) and normalizing them, we obtain ${D}_{{\rm{KL}}}=(2.0\pm 2.3)\times {10}^{-1}$. This comparison shows that the probability distributions of the spin configurations in the thousand cases are Boltzmann-like compared to general distributions. Note also that the instance dependence of *β* is small in the sense that the standard deviation is much smaller than the average.

We also simulate an instance of the four-spin Ising problem to check the case with more than two spins (*N* > 2). Since it is computationally hard to solve the quantum master equation in the four-spin case, we use the quantum-jump approach^34,35^, which is a Monte-Carlo simulation using a state vector, instead of a density matrix, and can provide equivalent results to the quantum master equation. The probability distribution obtained is also Boltzmann-like. (See Supplementary Information for details.)

### Quantum heating in dissipative QbMs

In this subsection, we first generalize the quantum heating to the case of multiple coupled nonlinear oscillators, and then explain that the generalized quantum heating results in the above Boltzmann distributions of the spin configurations.

Quantum heating is the heating process induced by quantum jumps due to dissipation in quasienergy levels of driven dissipative quantum nonlinear systems. This is well described by the balance equation derived from the quantum master equation^16,19^. We apply the balance-equation approach to the case of multiple coupled nonlinear oscillators.

In the previous studies on quantum heating, the Boltzmann distribution of quasienergies close to one of local minima of the Hamiltonian is derived analytically by deriving a local Hamiltonian by linearization around the chosen local minimum. In general, the effective temperature depends on the choice of the local minimum. However, this approach may not be useful for explaining the above Boltzmann distributions of the spin configurations, because each configuration corresponds to one of the local minima (see below) and therefore the explanation may need global information of the Hamiltonian. Hence we use numerically evaluated eigenvalues and eigenstates of the global (exact) Hamiltonian.

To explain this point in more detail, here we introduce the effective potential. The Hamiltonian in Eq. (2) is rewritten with the quadrature amplitudes ${x}_{i}=({a}_{i}+{a}_{i}^{\dagger })/2$ and ${y}_{i}=({a}_{i}-{a}_{i}^{\dagger })/2i$ as follows:

10 $$H=\hslash \sum _{i=1}^{N}{H}_{{\rm{K}}{\rm{P}}{\rm{O}}}^{(i)}-\hslash {\xi }_{0}\sum _{i=1}^{N}\sum _{j=1}^{N}{J}_{i,j}({x}_{i}{x}_{j}+{y}_{i}{y}_{j})+2\hslash {\xi }_{0}A(t)\sum _{i=1}^{N}{h}_{i}{x}_{i}$$

11 $${H}_{{\rm{K}}{\rm{P}}{\rm{O}}}^{(i)}=\hslash [\frac{K}{2}{({x}_{i}^{2}+{y}_{i}^{2})}^{2}+({\rm{\Delta }}-K)({x}_{i}^{2}+{y}_{i}^{2})-p(t)({x}_{i}^{2}-{y}_{i}^{2})+\frac{3K-4{\rm{\Delta }}}{8}]$$

The quadrature amplitudes are related to the dimensionless coordinate *Q* and momentum *P* in the literature^16,17,19^ as $Q=\sqrt{2\lambda }x$ and $P=\sqrt{2\lambda }y$, where *λ* is a dimensionless parameter introduced in the literature. The parameter *λ* and another dimensionless parameter *μ* in the literature^16,17,19^ are expressed with the present parameters as $\lambda =K/(2p)$ and $\mu =(K-{\rm{\Delta }})/p$. The effective potential ${V}_{{\rm{eff}}}({\bf{x}})$ is defined by setting all the momenta to zero and dropping constant terms in the Hamiltonian:

12 $${V}_{{\rm{e}}{\rm{f}}{\rm{f}}}({\boldsymbol{x}})=\hslash \sum _{i=1}^{N}[\frac{K}{2}{x}_{i}^{4}+({\rm{\Delta }}-K-p){x}_{i}^{2}]-\hslash {\xi }_{0}\sum _{i=1}^{N}\sum _{j=1}^{N}{J}_{i,j}{x}_{i}{x}_{j}+2\hslash {\xi }_{0}A\sum _{i=1}^{N}{h}_{i}{x}_{i}$$

**Figure 4:**
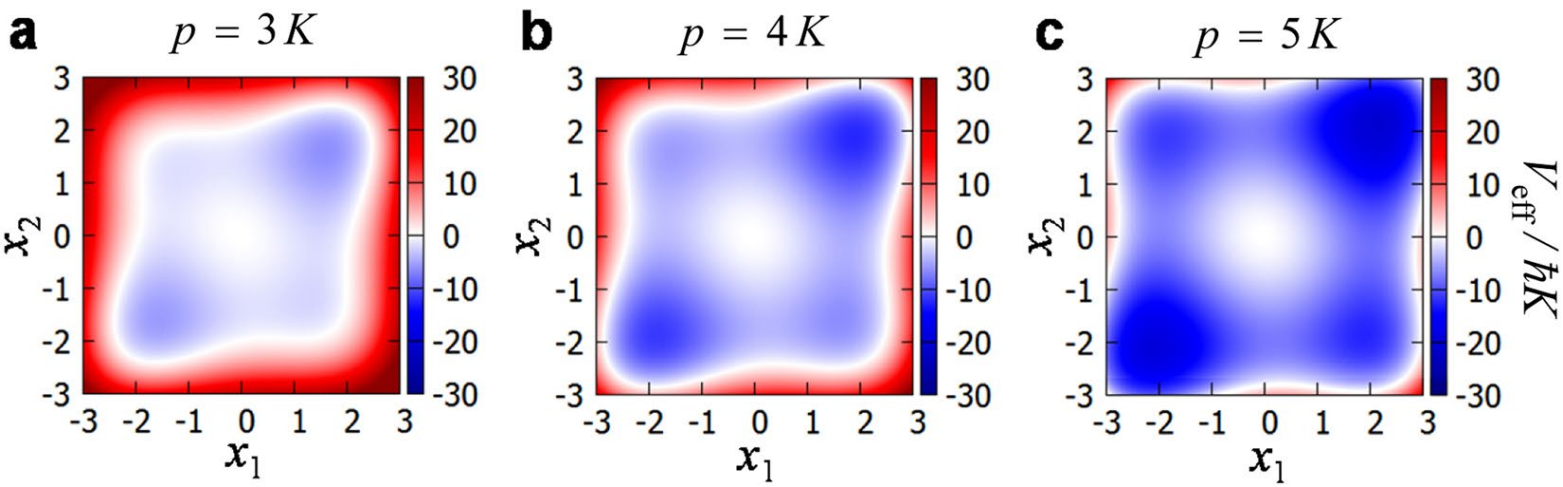
Effective potential. (**a–c**) The effective potentials defined by Eq. (12) with $p=3K$, 4*K*, and 5*K*, respectively, for the instance of the two-spin Ising model in Figs 1 and 2.

Figure 4 shows the effective potential for the two-spin instance in Fig. 2. The four local minima correspond to the four spin configurations. Note that the potential wells are deeper for larger *p*. This qualitatively explains why the convergence is slower for larger *p*_*f*_. In the previous studies on quantum heating, intrawell states in a chosen well have been used as the quasienergy states^16,17,19^. Instead, here we use the eigenstates of the global (exact) Hamiltonian as the quasienergy states.

Using the quasienergy states $\{|{E}_{n}\rangle \}$ as an orthonormal basis, the quantum master equation [Eq. (8)] becomes a system of ordinary differential equations of the density matrix ${\rho }_{m,n}=\langle {E}_{m}|\rho |{E}_{n}\rangle$. Note that in the equations for the diagonal elements $\{{\dot{\rho }}_{n,n}\}$, the terms for the unitary time evolution are cancelled out. Here the off-diagonal elements are disregarded assuming that the quasienergy separations, $|{E}_{m}-{E}_{n}|/\hslash$, are large compared to the decay rate *κ*, which is called the secular approximation^36^. Then we obtain the following balance equation with respect to the diagonal elements^16,19^:

13 $${\dot{\rho }}_{n,n}=2\kappa \sum _{i=1}^{N}\,\sum _{m=0}^{{\rm{\infty }}}({|{a}_{n,m}^{(i)}|}^{2}{\rho }_{m,m}-{|{a}_{m,n}^{(i)}|}^{2}{\rho }_{n,n})$$

where ${a}_{m,n}^{(i)}=\langle {E}_{m}|{a}_{i}|{E}_{n}\rangle$. Note that the diagonal element ${\rho }_{n,n}$ represents the probability that the system is in the quasienergy state $|{E}_{n}\rangle$.

A physical interpretation of the balance equation [Eq. (13)] is as follows. Dissipation induces quantum jumps corresponding to one-photon loss^34,35^. A quantum jump induced by an annihilation operator *a*_*i*_ changes $|{E}_{n}\rangle$ into ${a}_{i}|{E}_{n}\rangle$, and consequently causes the transition from $|{E}_{n}\rangle$ to $|{E}_{m}\rangle$ with probability proportional to ${|{a}_{m,n}^{(i)}|}^{2}$. Quantum heating is the heating process that originates from the transitions due to quantum jumps. Note that coherent states, which are often regarded as “most classical” states, are eigenstates of annihilation operators, and therefore the transitions due to quantum jumps do not occur for coherent states.

**Figure 5:**
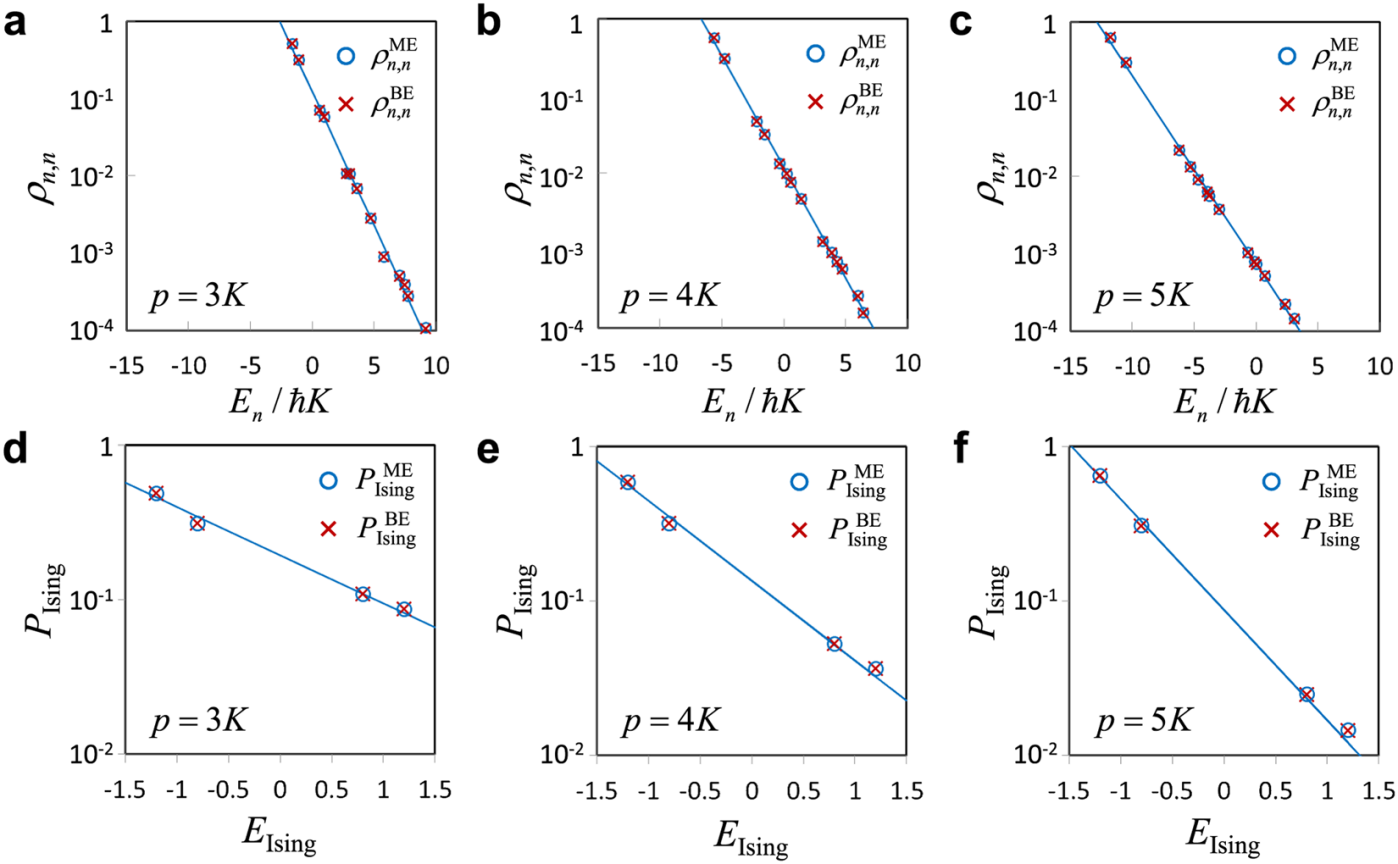
Quantum heating of dissipative QbM. The parameters of the two-spin Ising model are the same as that in Figs 1 and 2. (**a**–**c**) Probability distributions of the quasienergies ${E}_{n}$ defined by Eq. (14). ${\rho }_{n,\,n}^{{\rm{ME}}}$ (circles) and ${\rho }_{n,\,n}^{{\rm{BE}}}$ (crosses) correspond to the steady-state solutions of the master equation [Eq. (8)] with $\kappa =0.05K$ and the balance equation [Eq. (13)], respectively. The lines show exponential fits to $\{{\rho }_{n,\,n}^{{\rm{ME}}}\}$. (**d**–**f**) Probability distributions of the spin configurations. ${P}_{{\rm{Ising}}}^{{\rm{ME}}}({\boldsymbol{s}})$ (circles) and ${P}_{{\rm{Ising}}}^{{\rm{BE}}}({\boldsymbol{s}})$ (crosses) correspond to the steady-state solutions of the master equation with $\kappa =0.05K$ and the balance equation, respectively. The lines show the Boltzmann distribution fitting to ${P}_{{\rm{Ising}}}^{{\rm{ME}}}({\boldsymbol{s}})$.

The steady-state solution, $\{{\rho }_{n,n}^{{\rm{BE}}}\}$, of the balance equation is obtained by substituting ${\dot{\rho }}_{n,n}=0$ into Eq. (13) and solving the resultant equations under the constraint $\sum _{n}{\rho }_{n,n}=1$. We numerically evaluate the steady-state solution for the instance in Fig. 2. We also numerically evaluate the steady-state solution, $\{{\rho }_{n,n}^{{\rm{ME}}}\}$, of the master equation [Eq. (8)] with $\kappa =0.05K$ by using the simulation results at the final time in Fig. 2. These results are shown in Figs. 5a–5c. In Figs. 5a–5c, the quasienergies $\{{E}_{n}\}$ are defined as follows:

14 $${E}_{n}={\varepsilon }_{n}-2\times \hslash \frac{3K-4{\rm{\Delta }}}{8}$$

where {ε_*n*_} is the eigenvalues of the Hamiltonian in Eq. (2) and the constant term in Eq. (11) has been taken into account in order to compare the effective potential ${V}_{{\rm{eff}}}({\bf{x}})$ in Eq. (12).

The good agreement between the two steady-state solutions in Figs. 5a–5c means that the secular approximation mentioned above is good. The probability distributions are clearly Boltzmann-like (at least for low quasienergies). (The probabilities of higher quasienergies are lower bounded by numerical errors due to the truncation of the Hilbert space. See Supplementary Information for the details. However, this does not affect the following discussion because the probabilities of low quasienergies are dominant for the probabilities of the spin configurations.) This result suggests that the quasienergies of coupled nonlinear oscillators obey the Boltzmann distribution due to quantum heating. It is also remarkable that the Boltzmann distribution holds not only for negative quasienergies (near the bottoms of the potential wells) but also for positive quasienergies (above the potential barriers). This cannot be explained by the previous approach based on linearization around one of local minima. (As mentioned above, the quasienergy distribution deviates from the Boltzmann distribution near the top of the potential barrier in the case of single nonlinear oscillators^19^.) The explanation of the numerical results is left as an interesting open problem. In the following, we simply assume that the quasienergy distributions are Boltzmann-like.

The spin configuration probabilities, ${P}_{{\rm{Ising}}}^{{\rm{BE}}}({\boldsymbol{s}})$, for the steady-state solution of the balance equation are given by

15 $${P}_{{\rm{Ising}}}^{{\rm{BE}}}({\boldsymbol{s}})=\sum _{n}{\rho }_{n,n}^{{\rm{BE}}}{P}_{{\rm{Ising}}}^{(n)}({\boldsymbol{s}})$$

where ${P}_{{\rm{Ising}}}^{(n)}({\bf{s}})$ represent the spin configuration probabilities for the quasienergy state $|{E}_{n}\rangle$, that is,

16 $${P}_{{\rm{Ising}}}^{(n)}({\boldsymbol{s}})=\langle {E}_{n}|\prod _{i=1}^{N}{M}^{(i)}({s}_{i})|{E}_{n}\rangle $$

The comparison between ${P}_{{\rm{Ising}}}^{{\rm{BE}}}({\boldsymbol{s}})$ and ${P}_{{\rm{Ising}}}^{{\rm{ME}}}({\boldsymbol{s}})$ in Fig. 2b ($\kappa =0.05K$) is shown in Figs. 5d–5f. They are in excellent agreement with each other. Hence, the Boltzmann distribution of the spin configurations is well explained by the generalized quantum heating. This result can be understood under some approximations as follows. From the generalized quantum heating, the density operator of the steady state is approximately given by ${\rho }^{{\rm{SS}}}=\exp (-\beta ^{\prime} H)/Z^{\prime} (\beta ^{\prime} )$, where $\beta ^{\prime}$ is the inverse effective temperature and $Z^{\prime} (\beta ^{\prime} )={\rm{Tr}}[\exp (-\beta ^{\prime} H)]$ is the corresponding partition function. (The primes are used to distinguish them from the above ones for the spin configurations.) When the dissipation is sufficiently small, the state is approximately one of the stable oscillating states $|{\boldsymbol{s}}\rangle \,:=|{s}_{1}{\alpha }_{0}\rangle |{s}_{2}{\alpha }_{0}\rangle \cdots |{s}_{N}{\alpha }_{0}\rangle$ (${s}_{i}=\pm \,1$). By the classical approximation that the annihilation operator *a*_*i*_ is replaced by the amplitude ${s}_{i}{\alpha }_{0}$, we obtain [also see Eq. (4)]

17 $${P}_{{\rm{I}}{\rm{s}}{\rm{i}}{\rm{n}}{\rm{g}}}^{{\rm{B}}{\rm{E}}}({\boldsymbol{s}})\approx \langle {\boldsymbol{s}}|{\rho }^{{\rm{S}}{\rm{S}}}|{\boldsymbol{s}}\rangle \propto \exp [-2\hslash {\xi }_{0}{\alpha }_{0}^{2}\beta ^{\prime} {E}_{{\rm{I}}{\rm{s}}{\rm{i}}{\rm{n}}{\rm{g}}}({\boldsymbol{s}})]$$

Thus, the quantum heating leads to the Boltzmann distribution of the spin configurations. Moreover, this derivation indicates that $\beta =2\hslash {\xi }_{0}{\alpha }_{0}^{2}\beta ^{\prime}$. In the case of Figs. 5d–5f, the ratio $2\hslash {\xi }_{0}{\alpha }_{0}^{2}\beta ^{\prime} /\beta$ is close to unity, as expected. (The values are 1.11, 1.11, and 1.02 for $p=3K,\,4K,\,{\rm{and}}\,5K$, respectively.) This supports the above explanation.

It is also notable that the steady-state solution of the balance equation [Eq. (13)] is independent of *κ*. This can explain why *β* in Fig. 2e converges to a single value independent of *κ*. The decay-rate-independent *β* is a feature of quantum heating^17^.

## Discussion

We have found by numerical simulation that the probability distributions of the spin configurations in dissipative QbMs are Boltzmann-like. We have also explained that the Boltzmann distribution originates from the quantum heating generalized to multiple coupled nonlinear oscillators. The present work is based on numerical analysis. Further general and analytic treatment is desirable in future work. In particular, the Boltzmann distribution of quasienergies of the global Hamiltonian is remarkable from the viewpoint of the previous approach, and therefore detailed study on the quasienergy distribution is desirable.

It is expected to be feasible for current technologies to experimentally observe the quantum heating of a driven dissipative nonlinear oscillator network. The most promising physical system for this is superconducting circuits, because they have already been used for the experiments on quantum heating of a single nonlinear oscillator^18^, parametric oscillations^32^, and large Kerr effects^37,38^. It is also notable that the Boltzmann distributions of the spin configurations can be observed by the measurement of quadrature amplitudes with heterodyne detection^32^, which is easier than the direct measurement of quasienergy distributions.

The present result also broadens the potential applications of QbM, such as Boltzmann sampling. Since the simulation of Boltzmann sampling from the Ising model is computationally hard in general for current digital computers^39^, such a special-purpose machine for the Boltzmann sampling may be useful. In the case of Boltzmann machine learning, which is the most promising candidate among applications of the Boltzmann sampler, high connectivity between Ising spins is desirable because sparse connectivity degrades the performance of machine learning^23^. The implementation of high connectivity is one of the difficulties in experiments. The superconducting circuits proposed in refs^14,15^, which can be applied to the Ising problem with all-to-all connectivity, are promising approaches to this problem. The approach in ref.^15^ is based on the embedding technique proposed in ref.^40^, where a KPO does not directly correspond to an Ising spin and instead $N(N-1)/2$ KPOs represent *N* logical Ising spins. It is an interesting open problem whether or not such logical Ising spins also obey the Boltzmann distribution in the presence of dissipation.

Although it is an important and intriguing question whether or not the Boltzmann sampling using the dissipative QbM has some speedup over classical algorithms, this is beyond the scope of this paper. Nevertheless, our proposal is expected to open a new possibility for harnessing the behaviors of complex open quantum systems for practical applications. Thus, the present work is expected to trigger interdisciplinary research in the fields of quantum information science, nonequilibrium quantum systems, nonlinear dynamics, and artificial intelligence.

## Methods

### Fitting of the Boltzmann distribution to the simulation results

In the present work, the Boltzmann distribution is fitted to the simulation results of spin configuration probabilities by minimizing the Kullback-Leibler (KL) divergence *D*_KL_ between them, where the KL divergence between two probability distributions $\{{P}_{n}\}$ and $\{{Q}_{n}\}$ is defined as follows^31^:

18 $${D}_{{\rm{KL}}}(P||Q)=\sum _{n}{P}_{n}\,\mathrm{ln}\,\frac{{P}_{n}}{{Q}_{n}}$$

Note that the KL divergence is asymmetric with respect to the two distributions. In this paper, we choose the Boltzmann distribution as $\{{P}_{n}\}$ and the simulation results as $\{{Q}_{n}\}$.

## Electronic supplementary material

Supplementary Information
