# Supplementary material for: Boltzmann sampling from the Ising model using quantum heating of coupled nonlinear oscillators
Source: Sci Rep. 2018 May 8;8:7154. doi: 10.1038/s41598-018-25492-8 (PMC5940910; doi:10.1038/s41598-018-25492-8)
Supplement: Supplementary file 1 — Supplementary Information [file 41598_2018_25492_MOESM1_ESM.pdf]

Supplementary Information

Boltzmann sampling from the Ising model using quantum heating of coupled nonlinear oscillators

Hayato Goto,[1]* Zhirong Lin,[2] Yasunobu Nakamura[2,3]

[1]Frontier Research Laboratory, Corporate Research & Development Center, Toshiba Corporation, 1, Komukai-Toshiba-cho, Saiwai-ku, Kawasaki 212-8582, Japan.

[2]RIKEN Center for Emergent Matter Science (CEMS), Wako, Saitama 351-0198, Japan.

[3]Research Center for Advanced Science and Technology (RCAST), The University of Tokyo, Meguro-ku, Tokyo 153-8904, Japan.

Four-spin Ising problem

Here, we provide simulation results for an instance of the four-spin Ising problem in order to check whether the probability distributions of spin configurations in dissipative QbMs are also Boltzmann-like in the case with more than two spins ($N > 2$). As mentioned in the main text, it is computationally hard to solve the quantum master equation in the four-spin case. We therefore use the quantum-jump approach[34, 35].

The parameters are given by $J_{1,2} = J_{2,1} = 0.93406$, $J_{1,3} = J_{3,1} = 0.801243$, $J_{1,4} = J_{4,1} = 0.094465$, $J_{2,3} = J_{3,2} = -0.654609$, $J_{2,4} = J_{4,2} = 0.945369$, $J_{3,4} = J_{4,3} = 0.711242$, $h_1 = 0.429632$, $h_2 = 0.218071$, $h_3 = 0.395458$, and $h_4 = 0.195112$, which were chosen randomly from the interval $(-1, 1)$. The energy landscape of this instance is shown in Fig. S1a. The pump amplitude $p(t)$ follows Eq. (9) with $p_f = 6K$. The other parameters are set to $\Delta = 2K$, $\xi_0 = 0.2K$, and $\kappa = 0.02K$.

The simulation results are summarized in Fig. S1. The spin configuration probabilities $P_{\text{Ising}}^{\text{ME}}(\boldsymbol{s})$ are obtained by taking the average over 300 trajectories of the Monte-Carlo simulation. As shown in Fig. S1b, the probability distribution is Boltzmann-like.

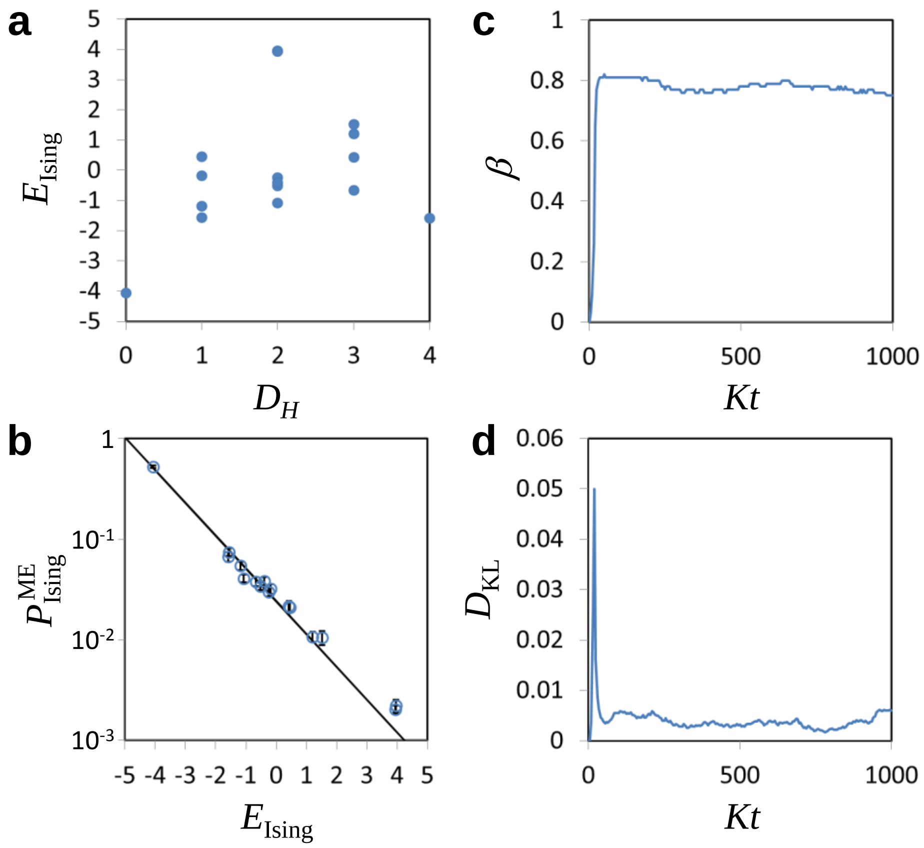

Fig. S1. Four-spin Ising model. (**a**) Energy landscape of the instance. The parameters are given in the text. (**b**) Distribution of the spin configuration probabilities $P^{\mathrm{ME}}_{\mathrm{Ising}}(\boldsymbol{s})$ at the final time ($t = 1000K^{-1}$). Error bars represent standard errors. The line shows the Boltzmann distribution fitting to the simulation results. (**c**) Inverse effective temperature β determined by fitting to the instantaneous probability distribution. (**d**) Kullbak-Leibler (KL) divergence D_{KL} minimized for the fitting in (c).

Detailed results of quasienergy distributions

Here, we provide the detailed results of the quasienergy distributions in Figs. 5a-5c in the main text. In Fig. S2, $N_{\max}$ denotes the photon number at which the Hilbert space for each KPO is truncated. The probabilities of high quasienergies are lower bounded. The lower bounds become lower for larger $N_{\max}$. Therefore, we conclude that this comes from numerical errors due to the truncation. On the other hand, from Figs. S2d-S2f, the probabilities of low quasienergies for $N_{\max} = 14$ are in good agreement with those for $N_{\max} = 16$. This ensures that the results for low quasienergies are reliable.

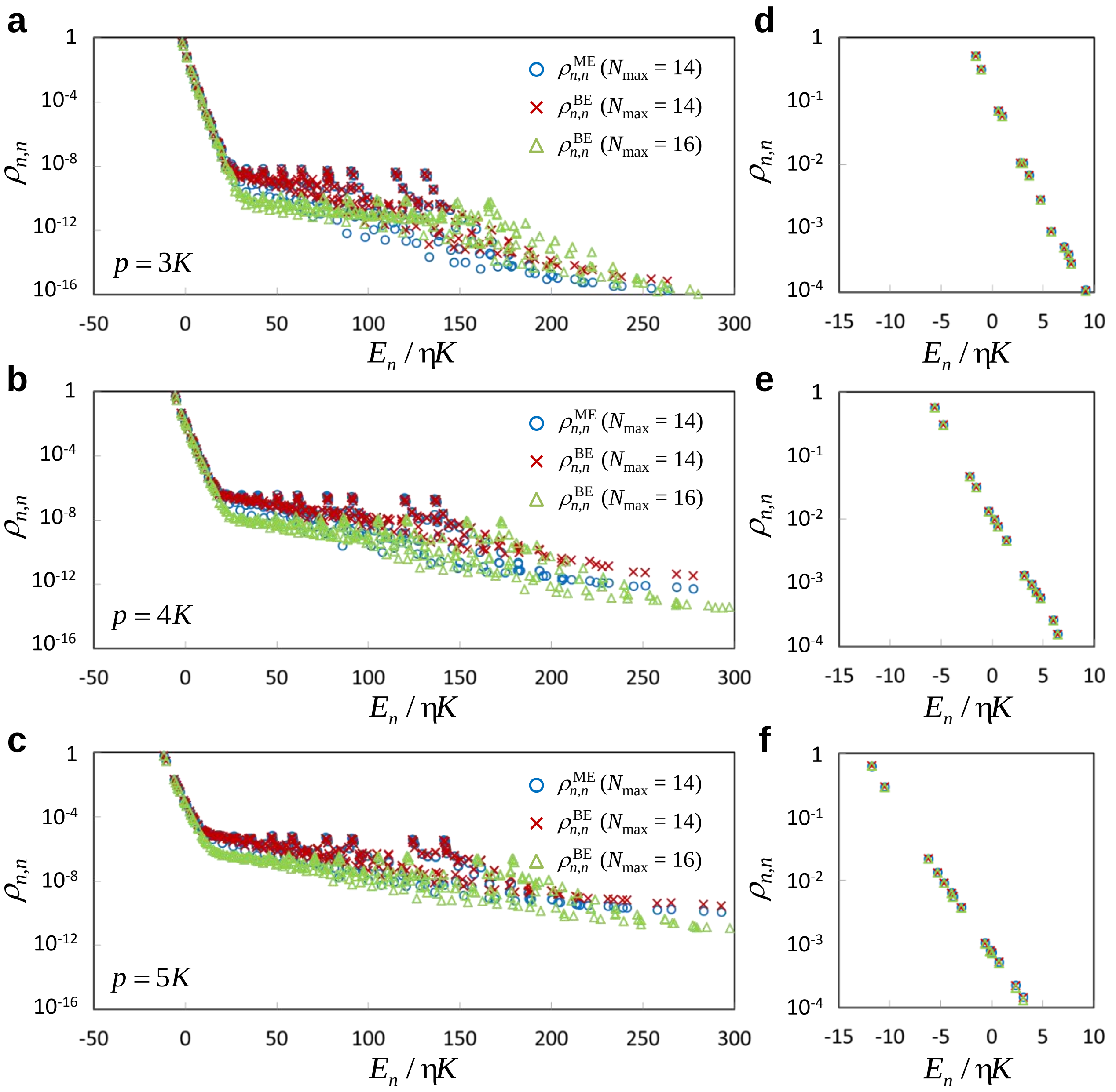

Fig. S2. Detailed quasienergy distributions in Fig. 5. (a-c) $\rho_{n,n}^{\mathrm{ME}}$ (circles) and $\rho_{n,n}^{\mathrm{BE}}$ (crosses and triangles) correspond to the steady-state solutions of the master equation with $\kappa = 0.05K$ and the balance equation, respectively. $N_{\max}$ denotes the photon number at which the Hilbert space for each KPO is truncated. **(d-f)** Magnifications of a-c, respectively.
